# Cardiovascular diseases among diffuse large B-cell lymphoma long-term survivors in Asia: a multistate model study

**DOI:** 10.1016/j.esmoop.2021.100363

**Published:** 2022-01-10

**Authors:** S.F. Lee, B.A. Vellayappan, L.C. Wong, C.L. Chiang, S.K. Chan, E.Y.-F. Wan, I.C.-K. Wong, P.C. Lambert, B. Rachet, A.K. Ng, M.A. Luque-Fernandez

**Affiliations:** 1 Department of Clinical Oncology, The University of Hong Kong, Hong Kong; 2 Department of Clinical Oncology, Tuen Mun Hospital, New Territories West Cluster, Hospital Authority, Hong Kong; 3 Department of Radiation Oncology, National University Cancer Institute, Singapore; 4 Centre for Safe Medication Practice and Research, Department of Pharmacology and Pharmacy, Li Ka Shing Faculty of Medicine, The University of Hong Kong, Hong Kong; 5 Department of Family Medicine and Primary Care, Li Ka Shing Faculty of Medicine, The University of Hong Kong, Hong Kong; 6 Laboratory of Data Discovery for Health (D24H), Hong Kong Science and Technology Park, Sha Tin, Hong Kong; 7 Research Department of Policy and Practice, School of Pharmacy, University College London, London, UK; 8 Biostatistics Research Group, Department of Health Sciences, University of Leicester, Leicester, UK; 9 Department of Medical Epidemiology and Biostatistics, Karolinska Institutet, Stockholm, Sweden; 10 Department of Non-Communicable Disease Epidemiology, ICON Group, London School of Hygiene and Tropical Medicine, London, UK; 11 Department of Radiation Oncology, Brigham and Women’s Hospital and Dana-Farber Cancer Institute, Harvard Medical School, Boston, USA; 12 Department of Non-Communicable Disease and Cancer Epidemiology, Instituto de Investigacion Biosanitaria de Granada (ibs.GRANADA), Andalusian School of Public Health, Granada, Spain

**Keywords:** non-Hodgkin’s lymphoma, diffuse large B-cell lymphoma, chemotherapy, radiotherapy, survival

## Abstract

**Background:**

We modeled the clinical course of a cohort of diffuse large B-cell lymphoma (DLBCL) patients with no prior cardiovascular diseases (CVDs) using a multistate modeling framework.

**Patients and methods:**

Data on 2600 patients with DLBCL diagnosed between 2000 and 2018 and had received chemotherapy with or without radiotherapy were obtained from a population-wide electronic health database of Hong Kong. We used the Markov illness-death model to quantify the impact of doxorubicin and various risk factors (therapeutic exposure, demographic, comorbidities, cardiovascular risk factors, and lifestyle factors which included smoking) on the clinical course of DLBCL (transitions into incident CVD, lymphoma death, and other causes of death).

**Results:**

A total of 613 (23.6%) and 230 (8.8%) of 2600 subjects died of lymphoma and developed incident CVD, respectively. Median follow-up was 7.0 years (interquartile range 3.8-10.8 years). Older ages [hazard ratio (HR) for >75 versus ≤60 years 1.88; 95% confidence interval (CI) 1.25-2.82 and HR for 61-75 versus ≤60 years 1.60; 95% CI 1.12-2.30], hypertension (HR 4.92; 95% CI 2.61-9.26), diabetes (HR 1.43; 95% CI 1.09-1.87), and baseline use of aspirin (HR 5.30; 95% CI 3.93-7.16) were associated with an increased risk of incident CVD. In a subgroup of anticipated higher-risk patients (aged 61-75 years, smoked, had diabetes, and received doxorubicin), we found that they remained on average 7.9 (95% CI 7.2-8.8) years in the DLBCL state and 0.1 (95% CI 0.0-0.4) years in the CVD state, if they could be followed up for 10 years. The brief time in the CVD state is consistent with the high chance of death in patients who developed CVD. Other causes of death have overtaken DLBCL-related death after about 5 years.

**Conclusions:**

In this Asian population-based cohort, we found that incident CVDs can occur soon after DLBCL treatment and continued to occur throughout survivorship. Clinicians are advised to balance the risks and benefits of treatment choices to minimize the risk of CVD.

## Highlights

• This is one of the largest studies describing life history path of DLBCL patients in Asia.
• Incident CVDs can develop soon after DLBCL treatment and continued to occur throughout survivorship.
• This prognostic model can be useful in individualized prognostic estimations for the survivorship outcome.
• We advise careful counseling and follow-up throughout lymphoma survivorship.

## Introduction

Diffuse large B-cell lymphoma (DLBCL) is the most common type of non-Hodgkin’s lymphoma (NHL) globally, constituting 30%-40% of all cases in different geographic regions. Effective modern therapeutic strategies have resulted in a 5-year median survival of 63.2% according to the US population-based data. However, a significant proportion of DLBCL survivors develop and die of treatment-related complications.^1^

The mainstay of therapeutic regimen for the treatment of patients with DLBCL includes rituximab, cyclophosphamide, doxorubicin, vincristine, and prednisone (R-CHOP) with or without radiotherapy (RT). This anthracycline-based chemotherapy regimen can increase the risk of cardiovascular sequelae; exposure to chest RT and preexisting cardiovascular risk factors may enhance the risk.^2^, ^3^, ^4^, ^5^, ^6^ The improvement in survival time should be interpreted in conjunction with long-term treatment-related toxicity. While lymphoma patients can experience different clinical events in the disease course,^1^ classical survival analyses estimate the probability to a single endpoint or perform separate analyses for each endpoint. However, these separate analyses do not describe the relations between different types of clinical events. In addition, available studies in survivors of aggressive NHL are often limited by missing key prognostic variables, such as treatment data.^5^, ^6^, ^7^, ^8^, ^9^, ^10^, ^11^, ^12^, ^13^, ^14^, ^15^

Limited data are available regarding the survival outcomes after the incidence of cardiovascular diseases (CVDs) among DLBCL survivors.^14^ The sequence of clinical events is important because a patient might have different prognoses after development of complications. Multistate models allow rich insights into complex disease pathways where a patient may experience intermediate events. Therefore, we aim to develop an illness-death multistate modeling approach to evaluate the prognostic factors affecting survival in DLBCL survivors, considering state transitions to CVD and death.

## Methods

### Study design, participants, data, and setting

**Figure 1:**
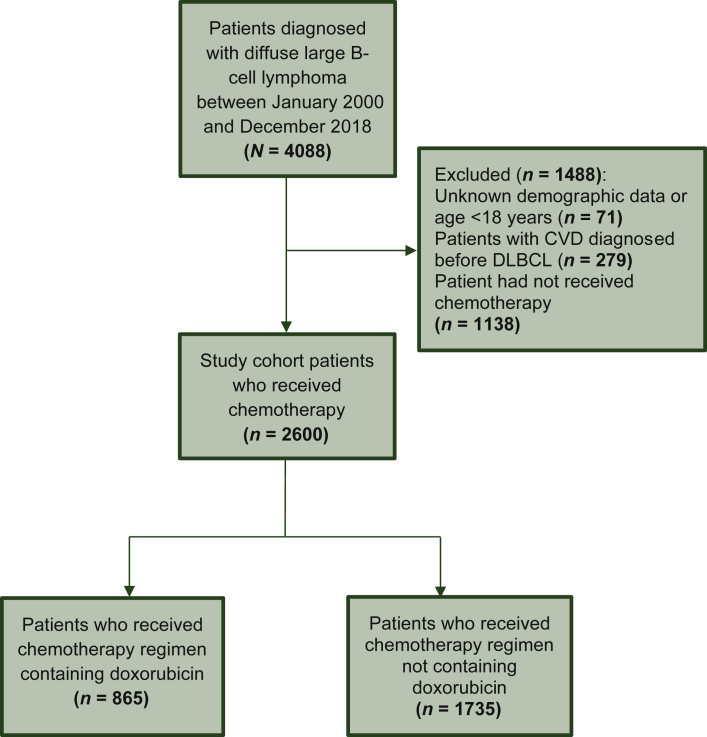
**CONSORT Diagram showing the inclusion and exclusion criteria (*N* = 2600), Hong Kong, 2000-2018.** CVD, cardiovascular disease; DLBCL, diffuse large B-cell lymphoma.

We conducted a population-based cohort study. Data were retrieved from the Clinical Data Analysis and Reporting System (CDARS; Supplementary Method S1, available at https://doi.org/10.1016/j.esmoop.2021.100363). Figure 1 shows the inclusion and exclusion criteria and the number of patients who finally constituted the study cohort.^16^ The cohort consisted of all DLBCL cases histologically diagnosed between 2000 and 2018 in Hong Kong. Patients were excluded if they (i) had unknown demographic data or aged <18 years (*N* = 71), (ii) developed CVD before DLBCL diagnosis (*N* = 279), and (iii) had not received chemotherapy for the DLBCL (*N* = 1138). Follow-up times for DLBCL cases (*N* = 2600) continued until absorbing states (lymphoma death or other causes of death), censor date 30 September 2019, or up to 15 years after baseline, whichever is earlier. The study protocol was approved by the Research Ethics Committee of the New Territories West Cluster, Hospital Authority, Hong Kong (reference no: NTWC/REC/19085).

### Outcomes and main exposures

#### Outcomes

The study outcomes included lymphoma deaths, other causes of death, and composite clinical events, which were the incident CVDs developed after diagnosis of DLBCL. The incident CVDs included ischemic heart disease, heart failure, cardiomyopathy, and stroke clinically diagnosed during inpatient hospital visits or as cause of death after the diagnosis of the lymphoma [International Classification of Diseases (ICD)-9 codes in Supplementary Table S1, available at https://doi.org/10.1016/j.esmoop.2021.100363].

#### Main exposure and other risk factors

The main exposure variables were sex, age at diagnosis, treatment exposures (use of chemotherapy, rituximab, and RT), cardiovascular risk factors, comorbidities (Supplementary Method S2, available at https://doi.org/10.1016/j.esmoop.2021.100363), and socioeconomic status. Cardiovascular risk factors included hypertension, dyslipidemia/hyperlipidemia, diabetes, and smoking.^17^^,^^18^ These medical conditions and smoking status were ascertained using a combination of ICD-9 codes, and the prescriptions of medications for these conditions (Supplementary Table S2 and Method S3, available at https://doi.org/10.1016/j.esmoop.2021.100363). We considered the use of medical financial assistance as a surrogate for low socioeconomic status.

### DLBCL treatment information

The treatment information included chemotherapy regimens (doxorubicin-based versus nondoxorubicin-based), use of rituximab, and RT. The absolute prescribed doses of doxorubicin were determined from pharmacy database. Patients who received doxorubicin were categorized by the absolute cumulative doses (≤500 or >500 mg, which is equivalent to ∼6 cycles of doxorubicin-containing regimen, or 300 mg/m^2^ assuming an average body surface area of 1.67 m^2^, a reasonable number based on local data).^19^

### Statistical analysis

Descriptive statistics for demographics, follow-up duration, and prevalence of characteristics were generated for the DLBCL survivors. Continuous variables were presented as medians with the minimum and maximum ranges, while categorical variables were presented as percentages. We reported the 2- and 5-year overall survival (OS) using the Kaplan–Meier method.

We then used a multistate modeling framework to study the natural course of patients with DLBCL and evaluated their mortality risk and survival experience accounting for the CVD competing risk up to 10 years of follow-up. The Markov illness-death model is a useful way of describing a journey in which an individual moves through a series of states in continuous time. We analyzed the course of DLBCL in two alive states:^20^ (i) alive after diagnosis of DLBCL, and (ii) alive after development of CVD; and two independent absorbing status: (i) DLBCL death and (ii) other causes of death (Supplementary Figure S1, available at https://doi.org/10.1016/j.esmoop.2021.100363). The arrows indicate the direction of possible transitions to four different states specified in boxes (i.e. states 1 to 4) (Supplementary Figure S1, available at https://doi.org/10.1016/j.esmoop.2021.100363). All patients began in the initial DLBCL state, which was defined as the time of lymphoma diagnosis, and could then move to a CVD state, or a dead state (lymphoma or other causes of death), and could also die after CVD (Supplementary Figure S1, available at https://doi.org/10.1016/j.esmoop.2021.100363). Details of the Markov illness-death model are in Supplementary Method S4, available at https://doi.org/10.1016/j.esmoop.2021.100363.

The multistate modeling was conducted using Stata version 16.1 (StataCorp, College Station, Texas, USA) and its *multistate* packages v4.3.0 and merlin v2.0.2.^21^^,^^22^ We provide the Stata code used to conduct the analysis in Supplementary Method S5, available at https://doi.org/10.1016/j.esmoop.2021.100363.

## Results

The characteristics of the DLBCL cohort (*N* = 2600) are detailed in Table 1. The median age at diagnosis for the DLBCL cohort was 63 years (interquartile range, 53-73 years); 56.0% were male. As of 30 September 2019, the median follow-up time from index date for the entire lymphoma survivor cohort was 7.0 years (interquartile range, 3.8-10.8 years). Overall, 848 patients died within 2 years of diagnosis (61.9% due to DLBCL), and 1103 patients died before the last day of follow-up (55.6% due to DLBCL).

**Table 1:** Characteristics of Diffuse Large B-Cell Lymphoma Patients, Hong Kong, 2000-2018 (*N* = 2600)

Characteristics	All lymphoma patients (*n* = 2600)	Lymphoma patients categorized by death (*n* = 1103)	
		Lymphoma death (*n* = 613)	Other causes of death (*n* = 490)
Patient’s factors			
Age at lymphoma diagnosis, year			
Median (range)	63 (18-97)	67 (19-95)	70 (18-97)
Sex, *n* (%)			
Male	1456 (56.0)	359 (58.6)	316 (64.5)
Female	1144 (44.0)	254 (41.4)	174 (35.5)
Race, *n* (%)			
Chinese	2484 (95.5)	594 (96.9)	475 (96.9)
Non-Chinese	116 (4.5)	19 (3.1)	15 (3.1)
Elevated LDH, *n* (%)	1336 (54.5)	415 (71.7)	272 (59.5)
RCS comorbidity scores, *n* (%)			
0	1710 (65.8)	417 (68.0)	253 (51.6)
1	664 (25.5)	159 (25.9)	179 (36.5)
≥2	226 (8.7)	37 (6.0)	58 (11.8)
Fee waiver recipients (surrogate for lower SES), *n* (%)	200 (7.7)	43 (7.0)	52 (10.6)
Hypertension, *n* (%)	1797 (69.1)	490 (79.9)	417 (85.1)
Diabetes mellitus, *n* (%)	598 (23.0)	170 (27.7)	140 (28.6)
Dyslipidemia/hyperlipidemia, *n* (%)	561 (21.6)	100 (16.3)	93 (19.0)
Smoker, *n* (%)	645 (24.8)	121 (19.7)	149 (30.4)
Aspirin use, *n* (%)	602 (23.2)	132 (21.5)	161 (32.9)
Treatment factors			
Chemotherapy, *n* (%)			
Regimens containing doxorubicin (>500 mg)	166 (6.4)	29 (4.7)	22 (4.5)
Regimens containing doxorubicin (≤500 mg)	699 (26.9)	173 (28.2)	123 (25.1)
Nondoxorubicin regimens	1735 (66.7)	411 (67.1)	345 (70.4)
Radiation, *n* (%)	308 (11.9)	120 (19.6)	78 (15.9)
Rituximab, *n* (%)	1980 (76.2)	395 (64.4)	334 (68.2)

Abbreviations: LDH, lactate dehydrogenase; RCS, Royal College of Surgeons; SES, socioeconomic status.

### Multistate illness-death model

**Figure 2:**
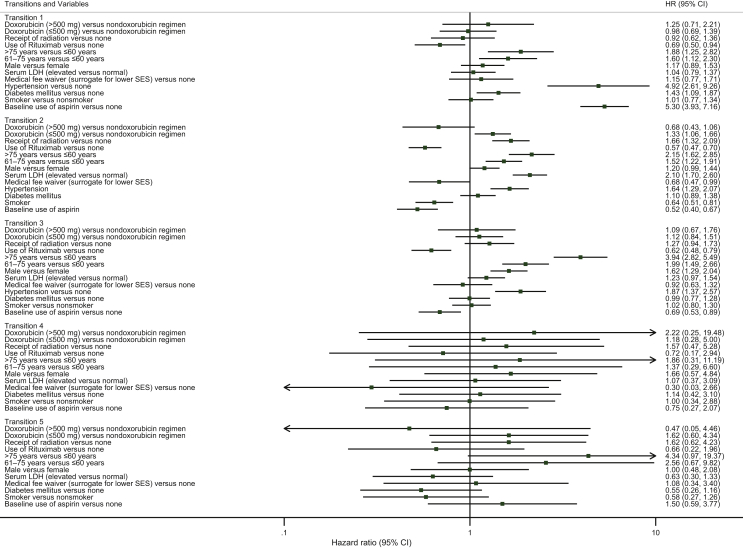
**Forest plot showing:the model estimates from the transition-specific models fitted to the diffuse large B-cell lymphoma, Hong Kong, 2000-2018 (*N* = 2600).** Transition 1: DLBCL diagnosis to CVD. Transition 2: DLBCL diagnosis to DLBCL death. Transition 3: DLBCL diagnosis to other causes of death. Transition 4: CVD to DLBCL death. Transition 5: CVD to other causes of death. Dyslipidemia/hyperlipidemia and comorbidity scores were not included in the model because of data scarcity in some transitions. Hypertension was not included in the models for transitions 4 and 5 because of lack of fit related to sparse data on hypertension and thus model convergence issues. CI, confidence interval; CVD, cardiovascular disease; DLBCL, diffuse large B-cell lymphoma; LDH, lactate dehydrogenase; SES, socioeconomic status.

The unadjusted 2- and 5-year OS were 72.2% [95% confidence interval (CI) 70.4% to 73.9%] and 62.4% (95% CI 60.4% to 64.3%), respectively. A total of 613 (23.6%) of 2600 patients died of lymphoma at 10 years of follow-up. The analyses based on multivariable flexible parametric hazard regression models showed that age [>75 versus ≤60 years; hazard ratio (HR) 1.88; 95% CI 1.25-2.82] and 61-75 versus ≤60 years (HR 1.60; 95% CI 1.12-2.30), cardiovascular risk factors [hypertension (HR 4.92; 95% CI 2.61-9.26), diabetes (HR 1.43; 95% CI 1.09-1.87)] and baseline use of aspirin (HR 5.30; 95% CI 3.93-7.16) were associated with a higher rate of CVD, while the use of rituximab (HR 0.69; 95% CI 0.50-0.94) decreased the rate (transition 1 in Figure 2 and Table 2).

**Table 2:** Model estimates from the transition-specific models fitted to the diffuse large B-cell lymphoma, Hong Kong, 2000-2018 (*N* = 2600)

	Multistate illness-death model^a^				
**Variables**	Transition 1^b^	Transition 2^c^	Transition 3^d^	Transition 4^e^	Transition 5^f^
	HR (95% CI)	HR (95% CI)	HR (95% CI)	HR (95% CI)	HR (95% CI)
Treatment factors					
Chemotherapy Doxorubicin (>500 mg) versus nondoxorubicin regimen	1.25 (0.71-2.21)	0.68 (0.43-1.06)	1.09 (0.67-1.76)	2.22 (0.25-19.48)	1.86 (0.47-7.29)
Doxorubicin (≤500 mg) versus nondoxorubicin regimen	0.98 (0.69-1.39)	1.33 (1.06-1.66)	1.12 (0.84-1.51)	1.18 (0.28-5.0)	1.62 (0.61-4.34)
Receipt of radiation	0.92 (0.62-1.36)	1.66 (1.32-2.10)	1.27 (0.94-1.73)	1.57 (0.47-5.28)	1.62 (0.62-4.23)
Use of rituximab	0.69 (0.50-0.94)	0.57 (0.47-0.70)	0.62 (0.49-0.79)	0.72 (0.18-2.94)	0.66 (0.22-1.96)
Patient factors					
Age at lymphoma diagnosis >75 years versus ≤60 years	1.88 (1.25-2.82)	2.15 (1.62-2.86)	3.94 (2.82-5.50)	1.86 (0.31-11.19)	4.34 (0.97-19.34)
61-75 years versus ≤60 years	1.60 (1.12-2.30)	1.52 (1.22-1.91)	1.99 (1.49-2.66)	1.38 (0.29-6.60)	2.56 (0.67-9.82)
Sex (male versus female)	1.17 (0.90-1.53)	1.20 (0.99-1.44)	1.62 (1.29-2.04)	1.66 (0.57-4.84)	1.00 (0.48-2.08)
Serum LDH (elevated versus normal)	1.04 (0.79-1.37)	2.10 (1.70-2.60)	1.23 (0.97-1.54)	1.07 (0.37-3.09)	0.63 (0.30-1.33)
Medical fee waiver (surrogate for lower SES)	1.15 (0.77-1.71)	0.68 (0.47-0.99)	0.92 (0.64-1.32)	0.30 (0.03-2.66)	1.08 (0.34-3.40)
Hypertension	4.92 (2.61-9.26)	1.64 (1.29-2.07)	1.87 (1.37-2.57)	—	—
Diabetes mellitus	1.43 (1.09-1.87)	1.10 (0.89-1.38)	1.00 (0.77-1.28)	1.14 (0.42-3.10)	0.55 (0.26-1.16)
Smoker	1.01 (0.77-1.34)	0.64 (0.51-0.81)	1.02 (0.80-1.30)	1.00 (0.35-2.88)	0.58 (0.27-1.26)
Baseline use of aspirin	5.30 (3.93-7.16)	0.52 (0.41-0.67)	0.69 (0.53-0.90)	0.75 (0.27-2.07)	1.50 (0.59-3.77)

CI, confidence interval; CVD, cardiovascular diseases; DLBCL, the diffuse large B-cell lymphoma; HR, hazard ratios; LDH, lactate dehydrogenase; SES, socioeconomic status.

a Dyslipidemia/hyperlipidemia and comorbidity scores were not included in the model because of data scarcity in some transitions. Hypertension was not included in the model for transition 4 and 5 due to model nonconvergence.

b Transition 1: DLBCL diagnosis to CVD.

c Transition 2: DLBCL diagnosis to DLBCL death.

d Transition 3: DLBCL diagnosis to other causes of death.

e Transition 4: CVD to DLBCL death.

f Transition 5: CVD to other causes of death.

Being older [>75 years (HR 2.15; 95% CI 1.62-2.86), 61-75 years (HR 1.52; 95% CI 1.22-1.91)], having received RT (HR 1.66; 95% CI 1.32-2.10), hypertension (HR 1.64; 95% CI 1.29-2.07), and elevated lactate dehydrogenase (HR 2.10; 95% CI 1.70-2.60) were associated with a higher rate of lymphoma death, while the use of rituximab (HR 0.57; 95% CI 0.47-0.70), having medical fee waiver (HR 0.68; 95% CI 0.47-0.99), being a smoker (HR 0.64; 95% CI 0.51-0.81), and baseline use of aspirin (HR 0.52; 95% CI 0.41-0.67) were associated with a lower risk of DLBCL-associated mortality for patients (transition 2 in Figure 2 and Table 2).

Patients had higher hazards of other causes of death if they were older [>75 years (HR 3.94; 95% CI 2.82-5.50), 61-75 years (HR 1.99; 95% CI 1.49-2.66)], being male sex (HR 1.62; 95% CI 1.29-2.04), and have hypertension (HR 1.87; 95% CI 1.37-2.57); the use of rituximab (HR 0.62; 95% CI 0.49-0.79) and baseline use of aspirin (HR 0.69; 95% CI 0.53-0.90) decreased the risk of other causes of death (transition 3 in Figure 2 and Table 2).

### Subgroup sensitivity analysis among 230 patients with incident CVD

**Figure 3:**
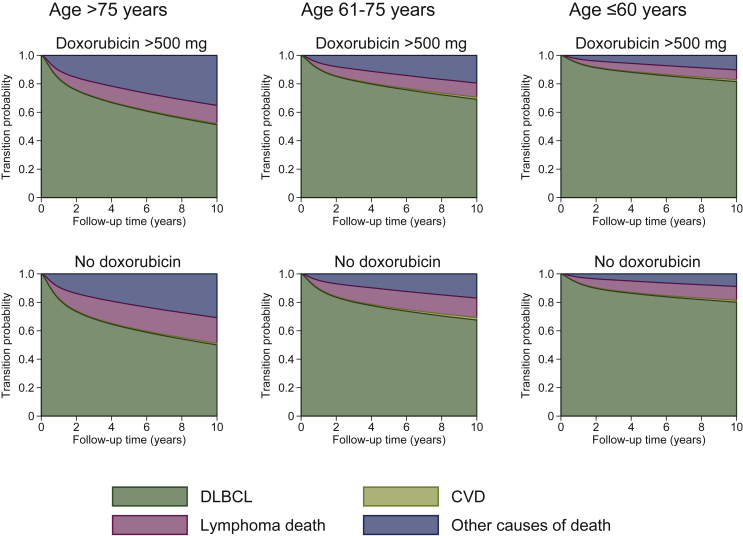
**Stacked graph showing probability from the illness-death model of being in each state among patients who were smokers, and had diabetes, varying across doxorubicin doses and ages, in Hong Kong, during 2000-2018 [613 lymphoma deaths (23.6%)].** CVD, cardiovascular disease; DLBCL, diffuse large B-cell lymphoma.

Among the entire study population of 2600 patients, 230 (8.8%) patients had incident CVD and 134 (6.4%) patients received doxorubicin dose >500 mg. We estimated the length of stay in each state, given a particular covariate pattern [based on age, doxorubicin, and cardiovascular risk factors (diabetes, smoking)]. Figure 3 and Supplementary Figure S2, available at https://doi.org/10.1016/j.esmoop.2021.100363 show the probability of being in each state for a patient who smoked, with diabetes, and received or not received doxorubicin.

Patients aged 61-75 years, smoked, had diabetes, and received doxorubicin on average remained 7.9 years (95% CI 7.2-8.8) in the DLBCL state and 0.1 years (95% CI 0.0-0.4) in the CVD state if they could be followed up for 10 years, before dying from lymphoma or from other causes. The brief time patients spent in the CVD state implies that those who developed CVD would have a high chance of further transition into death states. For this subgroup, other causes of death have overtaken DLBCL-related death after ∼5 years, as shown in Supplementary Figure S3, available at https://doi.org/10.1016/j.esmoop.2021.100363. Further sensitivity analysis showed that 59 patients had one of the major rheumatic diseases (Supplementary Table S2, available at https://doi.org/10.1016/j.esmoop.2021.100363). Chi-square test between their baseline aspirin use and the rheumatic diseases showed weak evidence of association (*P* = 0.176).

## Discussion

In a contemporary cohort of 2600 patients diagnosed with DLBCL without previous CVD, patients were found to have a trend of increased risk of transition to CVD and a higher risk of developing CVD and subsequent death if they had received higher doses (more cycles) of doxorubicin in primary treatment. Besides, we reported that age beyond 60 years and hypertension were associated with a higher risk of death and incident CVD, while elevated baseline serum lactate dehydrogenase (a surrogate for more advanced disease) was associated with a higher risk of lymphoma death. Previous studies showed that elderly patients may still benefit from anthracycline-based chemotherapy.^23^^,^^24^ However, the toxicities related to R-CHOP therapy are exacerbated with increasing age, functional disability, and comorbidity.^25^^,^^26^ Patients’ age might complicate the decision to use anthracycline-based chemotherapy, as shown in a large epidemiological study in the United States, in which patients aged >80 years were less common to receive R-CHOP regimen.^27^ Therefore it is crucial in having a comprehensive assessment of a patient’s fitness for anthracycline-based treatment before considering less toxic and potentially less effective alternatives.^28^

The presence of pre-existing cardiovascular risks factors such as diabetes and hypertension is found to be relevant for our DLBCL cohort. Hypertension was associated with increased risks of CVD and deaths. This finding corroborated with studies that reported comorbidity predicts for worse OS for adult patients with DLBCL^29^^,^^30^ and increased CVD risks among patients diagnosed with DLBCL or NHL,^6^^,^^14^ and cancer survivors in general.^12^^,^^31^ Baseline aspirin use was associated with a higher incident risk of CVD. This should be regarded as a secondary finding supplementary to the main results largely because of multiple comparisons in endpoints. Aspirin could be prescribed for other medical conditions such as rheumatic diseases. However, the sensitivity analysis has shown weak association between baseline aspirin use and these diseases. We hypothesize that aspirin use at baseline is likely a surrogate for pre-existing higher cardiovascular risk. We suggest that proactive pretreatment screening for these risk factors, and vigorous monitoring of cardiac function during and after lymphoma treatment may be helpful.^31^^,^^32^ RT was found to be associated with a higher probability of transitions into lymphoma-related death. However, we did not have detailed RT information from our database, such as RT sites, dose fractionation, and indication. It is likely that patients who received RT had more advanced disease, such as bulky sites or partial response to chemotherapy.

In our analyses, many sociodemographic and clinical factors found to be significantly associated with (or with a trend to affect) DLBCL-specific mortality also were factors associated with all other causes of death. Available data and the current study findings suggest that clinicians need to consider these factors when optimizing therapy to increase survival and reduce adverse events.^29^^,^^30^

We acknowledge that our study has several limitations. Similar to other studies of electronic health registry, detailed information on disease and certain patient characteristics such as performance status, dietary pattern, levels of physical exercise, and RT dose fractionation and sites were unavailable.^14^^,^^33^ The data were potentially affected by confounding by indication. It is possible that patients with mild (and therefore uncaptured) medical comorbidities were less likely to receive doxorubicin and therefore were treated with other regimens. The lack of prognostic factors, such as those in the International Prognostic Index,^34^ and patient-related factors, in the CDARS data precluded the analysis of these factors on the outcomes and the influence of selection on treatment strategies. There was no precise information about the exact date for the treatment variables, that is, doxorubicin doses, rituximab, and RT. Therefore sicker patients dying before getting treatment might have introduced a potential immortal-time bias. However, in a related paper studying the same group of patients, sensitivity analysis using different landmark periods produced consistent results.^35^ Finally, in our determination of incident CVD events, we conservatively restricted the events to those diagnosed at hospital or death to capture the symptomatic and most severe cases. This approach may lead to underestimation of the true incidence of cardiotoxicity by not including milder forms of CVD events. However, this avoids misclassification related to diagnostic coding errors and uncertainty in the diagnosis of milder CVD events. Previous studies have demonstrated high coding accuracy in diagnosis, demographics, and medication code retrieval from CDARS.^36^, ^37^, ^38^ It is likely that we would have captured the majority of the CVDs diagnosed at the hospital and death, because patients with chronic diseases and serious complications are mostly managed in our heavily subsidized public health care system. Although these data limitations may cause bias toward the null, we still detected important associations between disease and treatment factors and the risk of CVD. In addition, to improve the robustness of the results, we have conducted sensitivity analysis and adjusted for the covariables that were retrievable from the database.

Despite these limitations, our study has strengths. This is one of the most updated and largest multistate model studies to investigate the survivorship of patients with DLBCL in Asia. Multistate model offers a framework to analyze data with intermediate states and/or multiple endpoints. We account for competing risks of DLBCL-specific and other causes of death within the same conceptual framework to minimize bias resulting from examining the interdependent events in isolation.^39^^,^^40^ We analyzed a reasonably large and homogeneous cohort in Hong Kong. This allowed us to adjust for multiple covariables using flexible parametric method within the multistate model. Knowledge regarding the factors for transition to the CVD events would provide clinicians with more specific information to use in the decision-making process and counseling. Some studies used chemotherapy claims data or the number of cycles as surrogate estimates for chemotherapy dose.^6^^,^^14^^,^^41^ However, chemotherapy dose reductions are common, especially in patients >75 years of age. We were able to categorize doxorubicin exposure by prescribed doses. In our sensitivity analysis, the result provides support for transitioning survivorship plans from a focus on lymphoma-related deaths to other causes beyond 5 years after treatment. Additional research using data with details regarding medication regimen and RT is required to better assess the impact of these therapies on survivorship.

### Conclusions

We conducted an Asian population-based analysis to study the clinical course of DLBCL patients with no prior CVD, and assessed the dose-dependent effect of doxorubicin on incident CVD events and survival outcomes. Through simultaneous adjustments for multiple covariates and intermediate events, we showed associations which are not directly visible with a classical regression model. We found that incident CVDs can occur soon after lymphoma treatment and continued to occur throughout the follow-up. Together, these findings highlight the importance of pretreatment screening for cardiovascular risk, careful balancing of the risks and benefits of doxorubicin, and minimizing the risk of CVD throughout survivorship.
