# Supplementary material for: Cardiovascular diseases among diffuse large B-cell lymphoma long-term survivors in Asia: a multistate model study
Source: ESMO Open. 2022 Jan 10;7(1):100363. doi: 10.1016/j.esmoop.2021.100363 (PMC8760397; doi:10.1016/j.esmoop.2021.100363)

Supplementary Method S1. Clinical Data Analysis and Reporting System (CDARS)

CDARS is an electronic health database operated by the Hospital Authority of Hong Kong. CDARS was created in 1995 for clinical audit and research purposes. The Hospital Authority is the sole public healthcare provider for primary, secondary, and tertiary care and covers approximately 90% of all secondary and tertiary care in Hong Kong, which has a population of around 7.5 millions.[1] Data, including demographics, diagnoses, hospitalizations, and causes, and dates of death, are recorded in CDARS. The International Classification of Diseases, Ninth Revision (ICD-9), was used for disease coding. These diagnosis codes in CDARS demonstrated high coding accuracy in diagnosing various medical conditions with positive and negative predictive values around 90% have been demonstrated in previous studies.[2-4] High-quality population-based studies have been conducted based on the data retrieved from CDARS.[2-6]

References:

1 Census and Statistics Department, The Government of the Hong Kong Special Administrative Region. Population, 2020. https://www.censtatd.gov.hk/hkstat/sub/so20.jsp. Accessed August 8, 2020.

2 Chan EW, Lau WC, Leung WK, et al. Prevention of Dabigatran-Related Gastrointestinal Bleeding With Gastroprotective Agents: A Population-Based Study. Gastroenterology. 2015;149:586-95.e3.

3 Chan EW, Lau WC, Leung WK, et al. Prevention of Dabigatran-Related Gastrointestinal Bleeding With Gastroprotective Agents: A Population-Based Study. Gastroenterology. 2015;149:586-95.e3.

4 Wong AYS, Root A, Douglas IJ, Chui CSL, Chan EW, Ghebremichael-Weldeselassie Y, et al. Cardiovascular outcomes associated with use of clarithromycin: population based study. BMJ. 2016;352:h6926.

5 Chiu SS, Lau YL, Chan KH, Wong WH, Peiris JS. Influenza-related hospitalizations among children in Hong Kong. N Engl J Med. 2002;347:2097-2103.

6 Lau WCY, Chan EW, Cheung C-L, et al. Association Between Dabigatran vs Warfarin and Risk of Osteoporotic Fractures Among Patients With Nonvalvular Atrial Fibrillation. JAMA. 2017;317:1151-1158.

Supplementary Method S2. Royal College of Surgeons (RCS) adaptation of the Charlson Comorbidity Index

We measured comorbidities before the lymphoma diagnosis using the Royal College of Surgeons (RCS) adaptation of the Charlson Comorbidity Index.[1] We removed some cardiovascular risk factors from the overall RCS score to prevent overlap. The remaining comorbid conditions in the Index (peripheral vascular disease, dementia, chronic pulmonary disease, rheumatic disease, liver disease, hemiplegia/paraplegia, renal disease, acquired immune deficiency syndrome/human immunodeficiency viral infection) were combined into the score.

Reference:

1 Brusselaers N, Lagergren J. The Charlson Comorbidity Index in Registry-based Research. Methods of information in medicine. 2017;56:401-406.

Supplementary Method S3. Determination of smoking status in CDARS

We used similar approaches as proposed by Poulsen et al to determine the smoking status because these data were not directed captured by CDARS.[1] Smoking was captured by the ICD-9 code of V15.82, while chronic obstructive pulmonary disease (ICD-9 codes: 491, 492, 496) was used as proxy of heavy smoking.[1]

Reference:

1 Poulsen AH, Christensen S, McLaughlin JK, Thomsen RW, Sorensen HT, Olsen JH, et al. Proton pump inhibitors and risk of gastric cancer: a population-based cohort study. Br J Cancer. 2009;100:1503-1507.

Supplementary Method S4. Multistate illness death model (MIDM)

During the study, patients may die without developing cardiovascular disease (1→3 or 4), develop incident cardiovascular disease (1→2) or death afterwards (2→3 or 4). Using the MIDM we assumed a cause-specific setting where causes for death from non-lymphoma and lymphoma are independent. All patients begin at the state of initial DLBCL diagnosis, and can then move to a dead state, and can also die after CVD. The Markov assumption implies that given the present information, the progression of patients to the next state is independent from their progression into the previous state. Thus, transition's probabilities, transitions rates, and length of stay (LOS) in each state were presented.

We modelled each transition between the states using a flexible parametric modelling approach allowing for the smooth function of the baseline hazard.[1] We included in the model as confounders previously identified risk factors having a statistically significant impact on the timing and rate of each clinical event.[2,3] For both MIDM we included doxorubicin chemotherapy, use of radiotherapy, use of rituximab, age, sex, SES, lactate dehydrogenase (LDH), RCS comorbidity score, and cardiovascular risk factors for the state transitions.

We used a general survival simulation algorithm to calculate transition probabilities, LOS in each state, their respective ratios, and the associated confidence intervals (CI) for specific covariate patterns, to illustrate the impact of changing covariate levels.[4] LOS is interpreted as the average duration patient with a covariate pattern spends in each state. Stacked transition probability plots were generated to show how risk evolved over time. In the stacked graphs all transition probability curves are plotted on top of each other; the difference between two curves indicates the probability of being in each state given a particular covariate pattern. By estimating the risk for covariates that define different populations (who have different survival times and characteristics), we identified specific poor-risk subgroups based on data for each of the independent causes of death (i.e., lymphoma death and other causes of death [non-lymphoma death]).

To validate the consistency of our modelling approach we tested in sensitivity analysis the multistate model on a specific group of patients particularly at higher risk of developing incident CVD (i.e., aged >60 years with cardiovascular risk factors who received doxorubicin >500mg as primary treatment). The findings allow us to assess the combined effect of multiple risk factors prior to initiation of treatment on outcomes, which might shed light on the importance of comorbidity assessment.

References:

1 Royston P, Lambert PC, eds. Flexible Parametric Survival Analysis Using Stata: Beyond the Cox Model: London, UK: Stata Press; 2011.

2 Armitage P, Berry G, Matthews JNS, eds. Statistical methods in medical research. 4th ed. Oxford, UK: Blackwell Science; 2002.

3 Geskus RB, LLC. CP. Data Analysis with Competing Risks and Intermediate States. New York, NY: Taylor & Francis; 2016.

4 Crowther MJ, Lambert PC. Parametric multistate survival models: Flexible modelling allowing transition-specific distributions with application to estimating clinically useful measures of effect differences. Statistics in medicine. 2017;36:4719-4742.

Supplementary Method S5. Statistical analysis codes

/***
Cardiovascular Diseases Among Diffuse Large B-Cell Lymphoma Long-Term Survivors in Asia: A Multistate Model Study
***/

/***
Authors:
Shing Fung Lee, Chi Leung Chiang, Sik Kwan Chan, Paul C Lambert, Bernard Rachet, Lea Choung Wong, Balamurugan A Vellayappan, Eric Yuk-Fai Wan, Ian Chi-Kei Wong, Andrea K. Ng, Miguel Angel Luque-Fernandez****/

/***
Copyright (c) 2021

Permission is hereby granted, free of charge, to any person obtaining a copy
of this software and associated documentation files (the "Software"), to deal
in the Software without restriction, including without limitation the rights
to use, copy, modify, merge, publish, distribute, sublicense, and/or sell
copies of the Software, and to permit persons to whom the Software is
furnished to do so, subject to the following conditions:
The above copyright notice and this permission notice shall be included in
all copies or substantial portions of the Software.

THE SOFTWARE IS PROVIDED "AS IS", WITHOUT WARRANTY OF ANY KIND, EXPRESS OR
IMPLIED, INCLUDING BUT NOT LIMITED TO THE WARRANTIES OF MERCHANTABILITY,
FITNESS FOR A PARTICULAR PURPOSE AND NON INFRINGEMENT. IN NO EVENT SHALL THE
AUTHORS OR COPYRIGHT HOLDERS BE LIABLE FOR ANY CLAIM, DAMAGES OR OTHER
LIABILITY, WHETHER IN AN ACTION OF CONTRACT, TORT OR OTHERWISE, ARISING FROM,
OUT OF OR IN CONNECTION WITH THE SOFTWARE OR THE USE OR OTHER DEALINGS IN
THE SOFTWARE.

```
Bug reports: leesf at hku.hk
***/

describe
gen agecat = age
recode agecat 0/60=1 61/150=2

// Generate the overall survival time
generate lastfu_os = mdy(9,30,2019) // Administrative right censor
format lastfu_os %tdCCYY-NN-DD
replace lastfu_os = death_date if death==1 & death_date!=.
gen os = lastfu_os - lymphoma_date

//generate the time to CVD
gen CVD_time = composite_date - lymphoma_date
replace CVD_time = os if composite ==0

//generate "Lymphoma Death" (1=yes, 0=no)
gen lymphoma_death = 0
replace lymphoma_death = 1 if DeathCauseMainCauseICD10 == "C819" | DeathCauseMainCauseICD10 == "C820" |DeathCauseMainCauseICD10 == "C829" | DeathCauseMainCauseICD10 == "C833" | DeathCauseMainCauseICD10 == "C835" | DeathCauseMainCauseICD10 == "C837" | DeathCauseMainCauseICD10 == "C838" | DeathCauseMainCauseICD10 == "C839" | DeathCauseMainCauseICD10 == "C843" | DeathCauseMainCauseICD10 == "C844" | DeathCauseMainCauseICD10 == "C845" | DeathCauseMainCauseICD10 == "C851" | DeathCauseMainCauseICD10 == "C854" | DeathCauseMainCauseICD10 == "C859" | DeathCauseMainCauseICD10 == "C880" | DeathCauseMainCauseICD10 == "C910" | DeathCauseMainCauseICD10 == "C911" | DeathCauseMainCauseICD10 == "C920"  | DeathCauseMainCauseICD10 == "C921"  | DeathCauseMainCauseICD10 == "C950" | DeathCauseMainCauseICD10 == "C959"

gen nonlymphoma_death = 0
replace nonlymphoma_death = 1 if death ==1 & lymphoma_death==0

generate censordate =mdy(9,30,2019)
format censordate %tdCCYY-NN-DD
```

```
// generate the DURATION of last FU for Lymphoma DEATH. We censor events on Sep 30, 2019
gen lastfu_deathlymphoma_duration = censordate - lymphoma_date
replace lastfu_deathlymphoma_duration = death_date - lymphoma_date if lymphoma_death==1 & death_date!=.
gen lastfu_lymphoma = lymphoma_date + lastfu_deathlymphoma_duration

// generate the DURATION of last FU for NON-Lymphoma DEATH. We censor events on Sep 30, 2019
gen death_nonlymphoma_duration = censordate - lymphoma_date
replace death_nonlymphoma_duration = death_date - lymphoma_date if lymphoma_death==0  & death_date!=.
gen lastfu_nonlymphoma = lymphoma_date + lastfu_deathlymphoma_duration


replace composite = 1 if stroke_date!=.
replace composite_date = stroke_date if composite_date==. | composite_date>stroke_date
tab composite

// generate a composite risk factor
gen comb_risk = 0
replace comb_risk =1 if HT==1| DM==1 |smoker==1| dyslipid==1 | depression==1 |alcohol==1 |obesity==1
tab comb_risk

gen ldh_cat = ldh
recode ldh_cat 0/250 =0 251/20000= 1


//Baseline characteristics
summarize age, detail
tab sex
tab race
tab ldh_cat
tab rcs_score_gp
summarize os if death==0, detail
```

```
tab year_dx
tab pay
tab HT
tab DM
tab dyslipidemia
tab smoker
tab aspirin
tab dose_cat
tab RT
tab rituximab

summarize age if lymphoma_death ==1, detail
tab sex if lymphoma_death ==1
tab race if lymphoma_death ==1
tab ldh_cat if lymphoma_death ==1
tab rcs_score_gp if lymphoma_death ==1
summarize os if lymphoma_death ==1, detail
tab year_dx if lymphoma_death ==1
tab pay if lymphoma_death ==1
tab HT if lymphoma_death ==1
tab DM if lymphoma_death ==1
tab dyslipid if lymphoma_death ==1
tab smoker if lymphoma_death ==1
tab aspirin if lymphoma_death ==1
tab dose_cat if lymphoma_death ==1
tab RT if lymphoma_death ==1
tab rituximab if lymphoma_death ==1

summarize age if nonlymphoma_death ==1, detail
tab sex if nonlymphoma_death ==1
tab race if nonlymphoma_death ==1
tab ldh_cat if nonlymphoma_death ==1
tab rcs_score_gp if nonlymphoma_death ==1
summarize os if nonlymphoma_death ==1, detail
tab year_dx if nonlymphoma_death ==1
tab pay if nonlymphoma_death ==1
tab HT if nonlymphoma_death ==1
tab DM if nonlymphoma_death ==1
```

```
tab dyslipid if nonlymphoma_death ==1
tab smoker if nonlymphoma_death ==1
tab aspirin if nonlymphoma_death ==1
tab dose_cat if nonlymphoma_death ==1
tab RT if nonlymphoma_death ==1
tab rituximab if nonlymphoma_death ==1



// Overall survival in multistate model

// generate a "composite risk factor"
gen comb_risk2 = 0
replace comb_risk2 =1 if HT==1| DM==1 | dyslipid==1
tab comb_risk2

// generate age categories
gen age_cat2 = age
recode age_cat2 0/60=1 61/75=2 76/150=3


preserve

msset, id(id) states(death) times(os) //

//gen exitdate= _start + 365.25*10        // exit date of analysis is 10 years.
format exitdate %td
stset _stop, enter(_start) exit(time exitdate) failure(_status==1) scale(365.25)
sts list        //    2-year OS 71.8% (95% CI = 69.9%-73.6%), 5 years = 62.3% (95%CI
60.2%-64.3%)


drop if dose_cat==0


gen dodi9 = lymphoma_date + 270          // set up a landmark period of 9 months from
"lymphoma_date"
format dodi9 %td
```

```
gen dodi1exit= dodi9 + 365.25*10        // exit date of analysis is 10 years.
format dodi1exit %td

quietly tab dose_cat, generate(dose_cat)          // dose_cat 1 = chemo without
doxorubicin, 2 = chemo with <500mg doxorubicin, 3 = chemo with >500mg
doxorubicin
quietly tab rcs_score_gp, generate(rcs_score_gp)          //rcs_score_gp = comorbidity
score
quietly tab year_dx, generate(year_dx)                    // year of diagnosis

stset lastfu, failure(composite==1) origin(time dodi9) entry(time dodi9) exit(time
dodi1exit) id(id) scale(365.25)
sts graph, by (age_cat2)


restore




//Lymphoma death//
preserve

//wide format (before msset)
list id CVD_time composite lastfu_deathlymphoma_duration lymphoma_death if
id==1 | id==320| id==1581, sepby(id)

//use msset to reshape our wide dataset into the stacked format, with a row for each
transition of which a patient is at risk for
msset, id(id) states(composite lymphoma_death nonlymphoma_death)
times(CVD_time lastfu_deathlymphoma_duration death_nonlymphoma_duration) //
"composite" = composite CVD endpoint: heartfailure, ischemic heart disease,
cardiomyopathy, and stroke. //

//long format (after msset)
list id _start _stop _from _to _status _trans if id==1 | id==320| id==1581, noobs

mat tmat = (.,1,2,3\.,.,4,5\.,.,.,.\.,.,.,.)
mat colnames tmat = diag cvd deaddlbcl deadother
```

```
mat rownames tmat = diag cvd deaddlbcl deadother
mat list tmat

logistic adriamycin sex age_cat2 rcs_score_gp comb_risk pay RT aspirin rituximab ldh_cat, nolog
predict double ps                    //ps = propensity score
gen double HAW = ((adriamycin == 1) / ps) + ((adriamycin == 0) / (1 - ps))         // Compute the inverse probability Treatment weights (IPTW)
summarize HAW, detail
keep if inrange(HAW, r(p05), r(p95))

stset _stop [pw=HAW], enter(_start) failure(_status==1) scale(365.25)

//create dummy indicator variables from factor variables
quietly tab dose_cat, generate(dose_cat)
quietly tab rcs_score_gp, generate(rcs_score_gp)
quietly tab year_dx, generate(year_dx)
quietly tab age_cat2, generate(agecat)

gen dose_cattest = dose_cat
recode dose_cattest 3=2
quietly tab dose_cattest, generate(dose_cattest)



tab _trans4 HT
tab _trans5 HT
tab _trans4 DM
tab _trans5 DM
tab _trans4 dyslipid
tab _trans5 dyslipid
tab _trans4 smoker
tab _trans5 smoker
tab _trans4 rcs_score_gp
tab _trans5 rcs_score_gp
tab _trans4 pay
tab _trans5 pay
```

```
//Transition 1
stmerlin dose_cat3 dose_cat2 sex agecat3 agecat2 RT rituximab aspirin HT DM smoker ldh_cat pay if _trans1==1, dist(rp) df(3)
estimates store m1

//Transition 2
stmerlin dose_cat3 dose_cat2 sex agecat3 agecat2 RT rituximab aspirin HT DM smoker ldh_cat pay if _trans2==1, dist(rp) df(3)
estimates store m2

//Transition 3
stmerlin dose_cat3 dose_cat2 sex agecat3 agecat2 RT rituximab aspirin HT DM smoker ldh_cat pay if _trans3==1, dist(rp) df(3)
estimates store m3

//Transition 4
stmerlin dose_cat3 dose_cat2 sex agecat3 agecat2 RT rituximab aspirin DM smoker ldh_cat pay if _trans4==1, dist(rp) df(3)
estimates store m4

//Transition 5
stmerlin dose_cat3 dose_cat2 sex agecat3 agecat2 RT rituximab aspirin DM smoker ldh_cat pay if _trans5==1, dist(rp) df(3)
estimates store m5

range temptime 0 10 500

predictms, transmatrix(tmat) models(m1 m2 m3 m4 m5) probability at1(dose_cat3 1) timevar(temptime) ci aj

list _prob_* temptime in 1/5, noobs ab(20)
list _prob_* if temptime==10, noobs ab(20)
list temptime if _prob_at1_1_4 >= _prob_at1_1_3, noobs ab(20)


// stacked graph - figure 3
predictms, transmatrix(tmat) models(m1 m2 m3 m4 m5) probability at1(dose_cat3 1 agecat3 1 DM 1 smoker 1) timevar(temptime)
```

```
list _prob_* temptime in 1/5, noobs ab(20)
list _prob_* if temptime==10, noobs ab(20)

graphms
graph save "lymphoma death_highdose.gph", replace

predictms, transmatrix(tmat) models(m1 m2 m3 m4 m5) probability at1(dose_cat1 1 agecat3 1 DM 1 smoker 1) timevar(temptime)
list _prob_* temptime in 1/5, noobs ab(20)
graphms
graph save "lymphoma death_noadria.gph", replace

predictms, transmatrix(tmat) models(m1 m2 m3 m4 m5) probability at1(dose_cat3 1 agecat2 1 DM 1 smoker 1) timevar(temptime)
list _prob_* temptime in 1/5, noobs ab(20)
graphms
graph save "lymphoma death_highdose1.gph", replace

predictms, transmatrix(tmat) models(m1 m2 m3 m4 m5) probability at1(dose_cat1 1 agecat2 1 DM 1 smoker 1) timevar(temptime)
list _prob_* temptime in 1/5, noobs ab(20)
graphms
graph save "lymphoma death_noadria1.gph", replace

predictms, transmatrix(tmat) models(m1 m2 m3 m4 m5) probability at1(dose_cat3 1 agecat1 1 DM 1 smoker 1) timevar(temptime)
list _prob_* temptime in 1/5, noobs ab(20)
graphms
graph save "lymphoma death_highdose2.gph", replace

predictms, transmatrix(tmat) models(m1 m2 m3 m4 m5) probability at1(dose_cat1 1 agecat1 1 DM 1 smoker 1) timevar(temptime)
list _prob_* temptime in 1/5, noobs ab(20)
graphms
graph save "lymphoma death_noadria2.gph", replace

graph combine "C:\Users\user\Dropbox\MALF_LEE\STUDIES\Cardiovascular Disease Risk in DLBCL Survivors\Multi-state Analysis for DLBCL &
```

```
CVD\Analysis\lymphoma death_highdose.gph" "C:\Users\user\Dropbox\MALF_LEE\STUDIES\Cardiovascular Disease Risk in DLBCL Survivors\Multi-state Analysis for DLBCL & CVD\Analysis\lymphoma death_noadria.gph", name("lymphoma_death_figure_3_1", replace) ycommon rows(2)
graph save "lymphoma death_figure_3_1.gph", replace

graph combine "C:\Users\user\Dropbox\MALF_LEE\STUDIES\Cardiovascular Disease Risk in DLBCL Survivors\Multi-state Analysis for DLBCL & CVD\Analysis\lymphoma death_highdose1.gph" "C:\Users\user\Dropbox\MALF_LEE\STUDIES\Cardiovascular Disease Risk in DLBCL Survivors\Multi-state Analysis for DLBCL & CVD\Analysis\lymphoma death_noadria1.gph", name("lymphoma_death_figure_3_2", replace) ycommon rows(2)
graph save "lymphoma death_figure_3_2.gph", replace

graph combine "C:\Users\user\Dropbox\MALF_LEE\STUDIES\Cardiovascular Disease Risk in DLBCL Survivors\Multi-state Analysis for DLBCL & CVD\Analysis\lymphoma death_highdose2.gph" "C:\Users\user\Dropbox\MALF_LEE\STUDIES\Cardiovascular Disease Risk in DLBCL Survivors\Multi-state Analysis for DLBCL & CVD\Analysis\lymphoma death_noadria2.gph", name("lymphoma_death_figure_3_3", replace) ycommon rows(2)
graph save "lymphoma death_figure_3_3.gph", replace

grc1leg2 "C:\Users\user\Dropbox\MALF_LEE\STUDIES\Cardiovascular Disease Risk in DLBCL Survivors\Multi-state Analysis for DLBCL & CVD\Analysis\lymphoma death_figure_3_1.gph" "C:\Users\user\Dropbox\MALF_LEE\STUDIES\Cardiovascular Disease Risk in DLBCL Survivors\Multi-state Analysis for DLBCL & CVD\Analysis\lymphoma death_figure_3_2.gph"
"C:\Users\user\Dropbox\MALF_LEE\STUDIES\Cardiovascular Disease Risk in DLBCL Survivors\Multi-state Analysis for DLBCL & CVD\Analysis\lymphoma death_figure_3_3.gph", cols(3)
graph save "lymphoma death_figure_3.gph", replace
```

//Probability of being in each state for a patient who is age >60 to 75, high dose doxorubicin, and having cardiovascular risk factors - figure S2
predictms, transmatrix(tmat) models(m1 m2 m3 m4 m5) probability at1(dose_cat3 1 agecat2 1 DM 1 smoker 1) timevar(temptime) ci aj

list _prob_* temptime in 1/5, noobs ab(20)
list _prob_* if temptime==10, noobs ab(20)

```
twoway    (line _prob_at1_1_1 temptime, color(green)) ///
          (rarea _prob_at1_1_1_lci _prob_at1_1_1_uci temptime, color(green%30)) ///
          , legend(order(1 "Probability" 2 "95% confidence interval") cols(1) ring(0) pos(11)) ///
          ylabel(,angle(h) format(%3.2f)) ///
          xtitle("Follow-up (years)") ytitle("Probability") ///
          name(Probability_1, replace) ///
          saving(probability_1_2, replace)

twoway    (line _prob_at1_1_2 temptime, color(red)) ///
          (rarea _prob_at1_1_2_lci _prob_at1_1_2_uci temptime, color(red%30)) ///
          , legend(order(1 "Probability" 2 "95% confidence interval") cols(1) ring(0) pos(11)) ///
          ylabel(,angle(h) format(%3.2f)) ///
          xtitle("Follow-up (years)") ytitle("Probability") ///
          name(Probability_2, replace) ///
          saving(probability_2_2, replace)

twoway    (line _prob_at1_1_3 temptime, color(blue)) ///
          (rarea _prob_at1_1_3_lci _prob_at1_1_3_uci temptime, color(blue%30)) ///
          , legend(order(1 "Probability" 2 "95% confidence interval") cols(1) ring(0) pos(11)) ///
          ylabel(,angle(h) format(%3.2f)) ///
          xtitle("Follow-up (years)") ytitle("Probability") ///
          name(Probability_3, replace) ///
          saving(probability_3_2, replace)

twoway    (line _prob_at1_1_4 temptime, color(yellow)) ///
          (rarea _prob_at1_1_4_lci _prob_at1_1_4_uci temptime, color(yellow%30))
```

```
///
		, legend(order(1 "Probability" 2 "95% confidence interval") cols(1) ring(0) pos(11)) ///
		ylabel(,angle(h) format(%3.2f)) ///
		xtitle("Follow-up (years)") ytitle("Probability") ///
		name(Probability_4, replace) ///
		saving(probability_4_2, replace)

graph combine "C:\Users\user\Dropbox\MALF_LEE\STUDIES\Cardiovascular Disease Risk in DLBCL Survivors\Multi-state Analysis for DLBCL & CVD\Analysis\probability_1_2.gph" "C:\Users\user\Dropbox\MALF_LEE\STUDIES\Cardiovascular Disease Risk in DLBCL Survivors\Multi-state Analysis for DLBCL & CVD\Analysis\probability_2_2.gph" "C:\Users\user\Dropbox\MALF_LEE\STUDIES\Cardiovascular Disease Risk in DLBCL Survivors\Multi-state Analysis for DLBCL & CVD\Analysis\probability_3_2.gph" "C:\Users\user\Dropbox\MALF_LEE\STUDIES\Cardiovascular Disease Risk in DLBCL Survivors\Multi-state Analysis for DLBCL & CVD\Analysis\probability_4_2.gph", ycommon rows(2)
graph save "lymphoma death_figure_4 (61-75).gph", replace



//Length of Stay (LOS) of being age 61-75, high dose doxorubicin, and having cardiovascular risk factors - figure S3
predictms, transmatrix(tmat) models(m1 m2 m3 m4 m5) probability at1(dose_cat3 1 agecat2 1 DM 1 smoker 1) timevar(temptime) los ci aj
list _los_* temptime in 1/5, noobs ab(20)
list _los_* if temptime==10, noobs ab(20)

twoway	(line _los_at1_1_1 temptime, color(green)) ///
		(rarea _los_at1_1_1_lci _los_at1_1_1_uci temptime, color(green%30)) ///
		, legend(order(1 "Length of stay" 2 "95% confidence interval") cols(1) ring(0) pos(11)) ///
		ylabel(,angle(h) format(%3.2f)) ///
		xtitle("Follow-up(years)") ytitle("Length of stay") ///
		name(LOS_1, replace) ///
```

```
saving(LOS_1_2, replace)

twoway (line _los_at1_1_2 temptime, color(red)) ///
	(rarea _los_at1_1_2_lci _los_at1_1_2_uci temptime, color(red%30)) ///
	, legend(order(1 "Length of stay" 2 "95% confidence interval") cols(1) ring(0) pos(11)) ///
	ylabel(,angle(h) format(%3.2f)) ///
	xtitle("Follow-up (years)") ytitle("Length of stay") ///
	name(LOS_2, replace) ///
	saving(LOS_2_2, replace)

twoway (line _los_at1_1_3 temptime, color(blue)) ///
	(rarea _los_at1_1_3_lci _los_at1_1_3_uci temptime, color(blue%30)) ///
	, legend(order(1 "Length of stay" 2 "95% confidence interval") cols(1) ring(0) pos(11)) ///
	ylabel(,angle(h) format(%3.2f)) ///
	xtitle("Follow-up (years)") ytitle("Length of stay") ///
	name(LOS_3, replace) ///
	saving(LOS_3_2, replace)

twoway (line _los_at1_1_4 temptime, color(yellow)) ///
	(rarea _los_at1_1_4_lci _los_at1_1_4_uci temptime, color(yellow%30)) ///
	, legend(order(1 "Length of stay" 2 "95% confidence interval") cols(1) ring(0) pos(11)) ///
	ylabel(,angle(h) format(%3.2f)) ///
	xtitle("Follow-up (years)") ytitle("Length of stay") ///
	name(LOS_4, replace) ///
	saving(LOS_4_2, replace)

list _los_* if temptime==10, noobs ab(20)

graph combine "C:\Users\user\Dropbox\MALF_LEE\STUDIES\Cardiovascular Disease Risk in DLBCL Survivors\Multi-state Analysis for DLBCL & CVD\Analysis\LOS_1_2.gph" "C:\Users\user\Dropbox\MALF_LEE\STUDIES\Cardiovascular Disease Risk in DLBCL Survivors\Multi-state Analysis for DLBCL & CVD\Analysis\LOS_2_2.gph" "C:\Users\user\Dropbox\MALF_LEE\STUDIES\Cardiovascular Disease Risk in DLBCL Survivors\Multi-state Analysis for DLBCL & CVD\Analysis\LOS_3_2.gph"
```

```
"C:\Users\user\Dropbox\MALF_LEE\STUDIES\Cardiovascular Disease Risk in DLBCL Survivors\Multi-state Analysis for DLBCL & CVD\Analysis\LOS_4_2.gph", ycommon rows(2)
graph save "lymphoma death_figure_6 (61-75).gph", replace

restore
```

Supplementary Table S1. Data retrieval criteria for cardiovascular diseases

**Cardiovascular diseases**	**ICD-9 codes**
Ischemic heart disease	410-413, 414.0, 414.8, 414.9, 429.7, V45.81, V45.82
Cardiomyopathy and heart failure	425, 402.01, 402.11, 402.91, 404.01, 404.03, 404.11, 404.13, 404.91, 404.93, 428, V42.1
Stroke	430, 431, 432, 433.01, 433.11, 433.21, 433.31, 433.81, 433.91, 434.01, 434.11, 434.91, 435, 436, 437.0, 437.1

Abbreviation: ICD-9, The International Classification of Diseases Ninth Revision

We used similar approaches as proposed by Poulsen et al to determine the smoking status because these data were not directed captured by CDARS.[1] Smoking was captured by the ICD-9 code of V15.82, while chronic obstructive pulmonary disease (ICD-9 codes: 491, 492, 496) was used as proxy of heavy smoking.[1]

Reference:

1 Poulsen AH, Christensen S, McLaughlin JK, Thomsen RW, Sorensen HT, Olsen JH, et al. Proton pump inhibitors and risk of gastric cancer: a population-based cohort study. Br J Cancer. 2009;100:1503-7.

Supplementary Table S2. ICD-9 codes and medications of the cardiovascular risk factors

**Cardiovascular risk Factors**	**ICD-9 codes**	**Medications**
Hypertension	401.X, 402.X, 403.X, 404.X, 405.X	Amlodipine, Diltiazem, Felodipine, Nifedipine, Verapamil, Atenolol, Bisoprolol Carvedilol, Labetalol, Metoprolol, Propranolol, Clonidine, Hydralazine, Losartan, Telmisartan, Valsartan, Bumetanide, Frusemide, Amiloride, Eplerenone, Spironolactone, Hydrochlorothiazide, Indapamide, Moduretic, Dyazide, Methyldopa, Doxazosin, Prazosin, Terazosin, Captopril, Enalapril, Lisinopril, Perindopril
Dyslipidemia	272.0, 272.1, 272.2, 272.3, 272.4	Atorvastatin, Rosuvastatin, Simvastatin, Fenofibrate, Gemfibrozil, Ezetimibe
Diabetes	249, 250.xx, 357.2, 362.0, 366.41, or 648.0	Insulin Neutral, Insulin Lispro, Insulin Aspart, Insulin Human, Insulin Lispro Human, Insulin Aspart Human Analog, Insulin Detemir, Insulin Isophane Human, Insulin Degludec, Insulin Glargine, Gliclazide, Glimepiride, Glipizide, Metformin, Alogliptin, Linagliptin, Sitagliptin, Dulaglutide, Exenatide, Liraglutide, Lixisenatide, Pioglitazone, Dapagliflozin, Empagliflozin, Acarbose
Depression	296.2, 296.3, 300.4, 311	Citalopram, Escitalopram, Fluoxetine, Fluvoxamine, Paroxetine, Sertraline, Venlafaxine, Desvenlafaxine, Duloxetine, Amitriptyline, Doxepin, Imipramine, Vortioxetine, Milnacipran, Mirtazapine, Mianserin, Trazodone
Chronic obstructive pulmonary disease	491, 492, 496	Beclomethasone, Budesonide +/- Formoterol, Fluticasone, Flutiform or equivalent, Relvar or equivalent, Seretide or equivalent, Trelegy or equivalent, Anoro or equivalent, Ultibro or equivalent, Spiolto or equivalent, Combivent or equivalent, Tiotropium, Ipratropium, Glycopyrronium, Indacaterol, Salbutamol, Montelukast, Roflumilast, Theophylline, Terbutaline
Smoking	V15.82, 491, 492, 496	Same as in chronic obstructive pulmonary disease

Alcoholism	291, 303, 305.0, 571.0, 571.1, 571.2, 571.3, 980.8, 980.9	-
Rheumatic diseases	710, 711, 712, 713, 714, 725	

Abbreviation: ICD-9, The International Classification of Diseases Ninth Revision

Supplementary Figure S1. Multistate illness-death model.

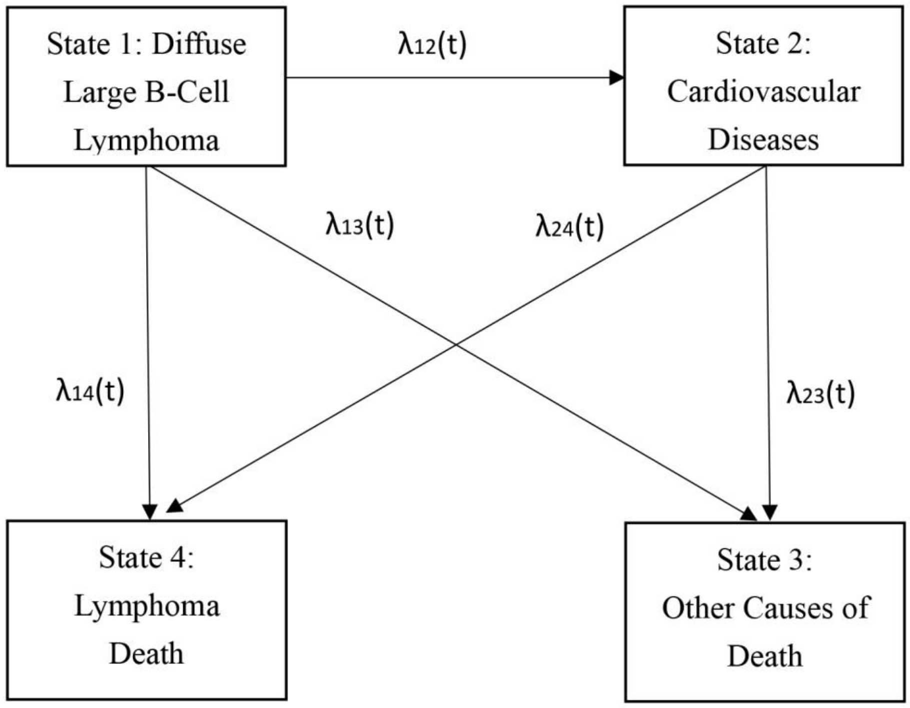

Supplementary Figure S2. Probability and 95% CI from the illness-death model. (A) DLBCL state, (B) CVD state, (C) DLBCL death state, and (D) other causes of death state for a patient aged 61–75, received doxorubicin, and being smoker and had diabetes, in Hong Kong, during 2000-2018 (613 lymphoma deaths [23.6%]). The light grey bands represent the 95% confidence interval bands.

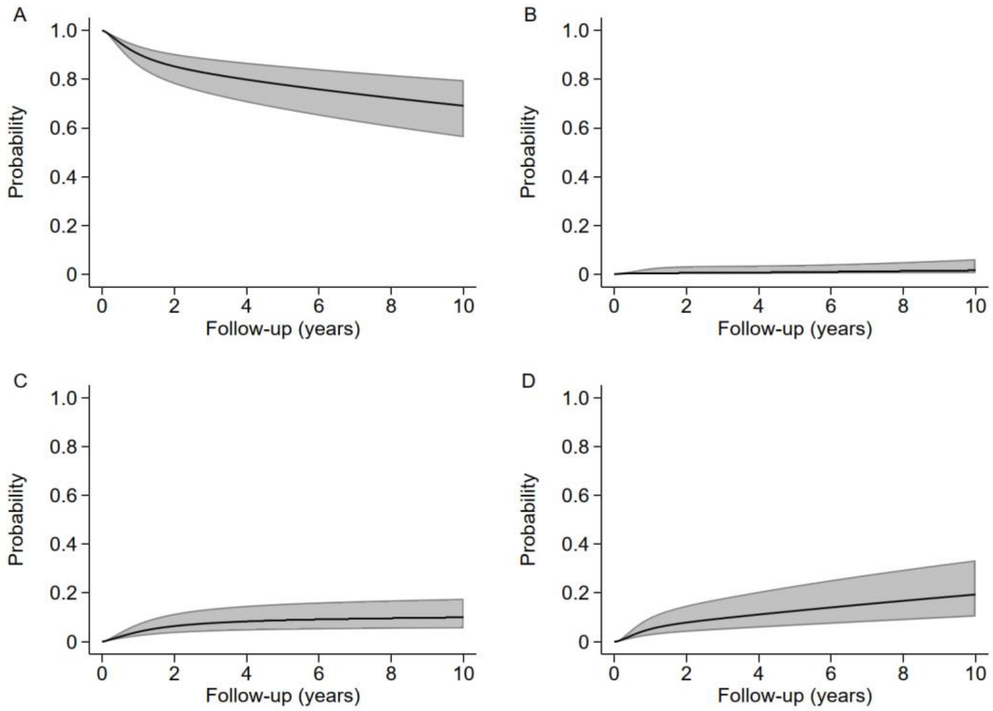

Supplementary Figure S3. Length of stay and 95% CI from the illness-death model. (A) DLBCL state, (B) CVD state, (C) DLBCL death state, and (D) other causes of death state for a patient aged 61–75 years, received doxorubicin, and being smoker and had diabetes, in Hong Kong, during 2000-2018 (613 lymphoma deaths [23.6%]). The light grey bands represent the 95% confidence interval bands.

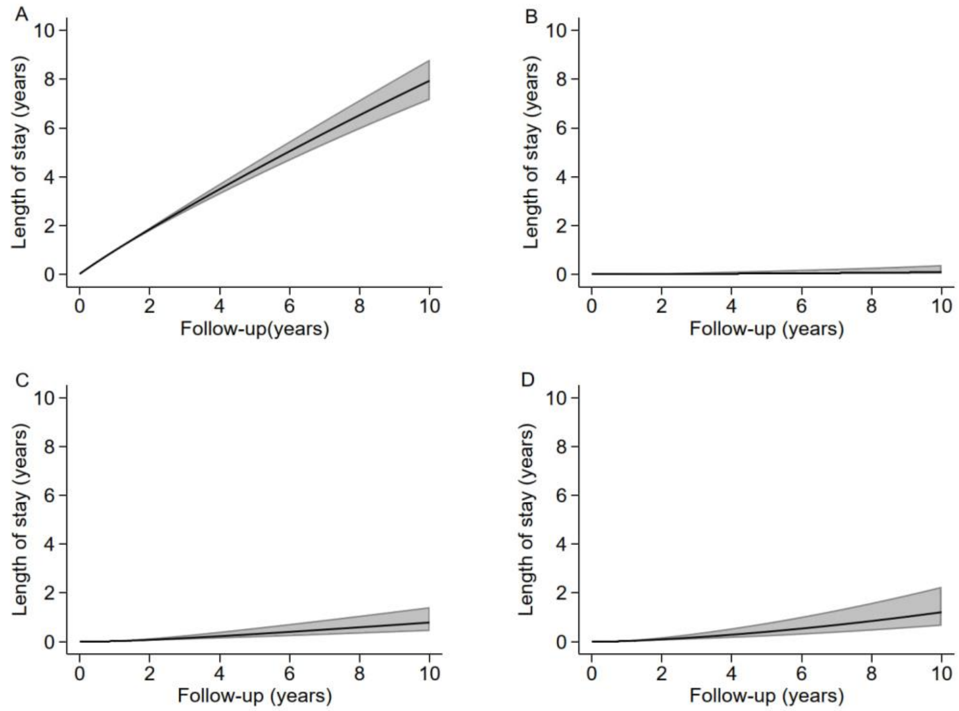

Supplement: Supplement [file mmc1.pdf]
